# Methodology for Studying Hypothalamic Regulation of Feeding Behaviors

**DOI:** 10.3390/mps7060086

**Published:** 2024-10-24

**Authors:** Julia B. Davenport, Ali D. Güler, Qi Zhang

**Affiliations:** 1 Department of Biology, University of Virginia, Charlottesville, VA 22904, USA; jbd2g@virginia.edu (J.B.D.);; 2 Program in Fundamental Neuroscience, Charlottesville, VA 22904, USA; 3 Department of Neuroscience, School of Medicine, University of Virginia, Charlottesville, VA 22903, USA

**Keywords:** feeding, methodology, hypothalamus

## Abstract

Continuous advances in neurological research techniques are enabling researchers to further understand the neural mechanisms that regulate energy balance. In this review, we specifically highlight key tools and techniques and explore how they have been applied to study the role of the hypothalamic arcuate nucleus in feeding behaviors. Additionally, we provide a detailed discussion of the advantages and limitations associated with each methodology.

## 1. Introduction

The ongoing obesity epidemic presents a significant global socioeconomic challenge, adversely affecting quality of life and contributing to widespread medical conditions such as type 2 diabetes, hypertension, and coronary heart disease [1,2,3]. Understanding the neural circuits that govern food intake and energy expenditure is crucial for uncovering how organisms maintain energy homeostasis, with far-reaching implications for public health and potential therapeutic interventions [1]. 

The arcuate nucleus (ARC), located at the base of the hypothalamus, serves as a critical hub for processing and integrating peripheral signals reflecting the body’s energy status [4]. It contains distinct populations of neurons with opposing effects on feeding: the orexigenic neurons that promote appetite, including the agouti-related peptide (AgRP) neurons [5,6,7,8], and the anorexigenic neurons that suppress it, represented by the pro-opiomelanocortin (POMC) neurons [9]. These neurons communicate with each other and other hypothalamic regions to regulate feeding behaviors and metabolism.

The ARC interacts with multiple hypothalamic regions to orchestrate feeding regulation. The AgRP neurons and POMC neurons in the ARC project to the paraventricular nucleus of the hypothalamus (PVH), the lateral hypothalamus (LH), ventromedial hypothalamus (VMH), and the dorsomedial hypothalamus (DMH) [1,10]. Stimulation of the AgRP neuron projections in the PVH or LH is sufficient to promote feeling [11], while deletion of the Neuropilin-2 receptor in POMC neurons disrupts their projections to the PVH and leads to weight gain [12].

Recent advancements in neuroscience have provided a multitude of techniques to study the neuronal mechanism underlying feeding behaviors. While traditional approaches such as lesion studies and pharmacological studies have provided important insights, modern methodologies including chemogenetics, optogenetics, and advanced imaging techniques offer enhanced benefits. These contemporary tools allow researchers to more precisely dissect the functional roles of hypothalamic circuits involved in feeding. This review aims to provide a comprehensive overview of the methodologies employed in hypothalamic feeding studies, highlighting the cutting-edge techniques. By examining the strengths, limitations, and applications of these methodologies, we seek to offer insights into how they contribute to our understanding of feeding behaviors and identify future directions for research in this vital area of neuroscience.

## 2. Manipulating the Activity of Feeding-Associated Neurons

### 2.1. Chemogenetics Using DREADDs

**Figure 1:**
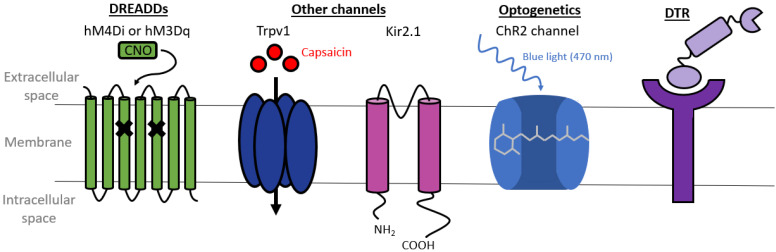
Tools to manipulate the activity of feeding-associated neurons.

To investigate the cellular basis of neurological functions, researchers employ chemogenetic technologies by engineering proteins, such as G protein-coupled receptors (GPCRs), to selectively interact with small molecules [13]. Among the most widely used engineered proteins are the Designer Receptors Exclusively Activated by Designer Drugs (DREADDs) [13], which harness GPCR signaling through recombinant receptors activated exclusively by synthetic ligands (Figure 1). The specificity that DREADDs are only activated by these designer drugs while unaffected by endogenous ligands enables precise spatial control of receptor activation through targeted expression using viral vectors. For example, by using Cre-mediated recombination to specifically express the excitable DREADDs receptor hM3Dq in AgRP neurons, the injection of clozapine-N-oxide (CNO) has been shown to elicit AgRP neuronal depolarization, which subsequently increased food intake and reduced energy expenditure [7]. Similarly, reciprocal expression of Cre-dependent hM3Dq in AgRP neurons and Dre-dependent inhibitory hM4Di in POMC neurons has been shown to increase food intake following CNO injection [14]. Interestingly, by activating a subpopulation of AgRP neurons co-expressing dopamine receptor D1 (Drd1) using a Cre- and Flp-dependent hM3Dq system is sufficient to promote feeding [15]. Despite these robust effects on food intake, neither activation nor inhibition of AgRP neurons affected glycemia, while activation led to significant impairment in insulin sensitivity [16]. Additionally, chemogenetic activation of AgRP neurons reduced anxiety in fed mice, and inactivation of these neurons diminished the anxiolytic effects induced by fasting [17,18].

The use of cre-dependent AAV-DREADDs in conjunction with transgenic animal models allows researchers to rapidly and reversibly regulate neuronal activity, thereby facilitating the investigation of mechanisms governing feeding and associated behaviors [7]. A critical advantage of this approach is that the same animal can serve as its own control, as it can undergo saline and CNO injections across its lifetime [13]. In comparison to other methods, such as optogenetics, DREADDs technology is preferable for evoking long-term activation of neurons as the effects of CNO could last 8–24 h following injection. 

However, chemogenetics has limitations, including restricted control over the valence and duration of neuronal manipulation. Evidence suggests that many receptors theoretically activated exclusively by synthetic ligands (RASSLs) exhibit high basal activity, leading to unexpected phenotypes even in the absence of ligand activation [13]. In addition, there are several limitations associated with the DREADDs ligand CNO. Firstly, CNO may take minutes to produce its desired effect, and has been reported to bind to alternative targets at concentrations required for DREADDs activation [19], resulting in less precise temporal control and potential off-target effects. Moreover, there are concerns about CNO’s rapid in vivo transformation. Evidence indicates that peripherally administered CNO does not cross the blood–brain barrier, and is metabolized into clozapine and N-desmethylclozapine (N-Des) within the brain [19]. Both clozapine and N-Des can influence various neurological functions, including alterations in glutamate and dopamine (DA) levels across multiple brain regions [20]. Novel DREADDs activators such as Compound 21, perlapine, and deschloroclozapine (DCZ) have been developed as alternatives to CNO and may address some of these issues [21,22,23]; however, thorough assessments of their potential side effects are still needed. Lastly, repeated use of the synthetic ligands for the DREADDs could result in receptor desensitization and downregulation [13]. Overall, while chemogenetic tools like DREADDs provide powerful means for manipulating neuronal activity, ongoing research and development are essential to address their limitations and enhance their efficacy.

### 2.2. Chemogenetics Using Other Channels

Another significant approach in chemogenetic manipulation involves using specific ion channels to control neural activity. Genetic manipulation of these channels enables researchers to induce either transient or chronic alterations in neural activity, allowing them to test how these molecular changes impact behavior. One example of this approach is to exclusively express the transient receptor potential cation channel subfamily V member 1 (Trpv1) (Figure 1) in AgRP neurons, as demonstrated by the AgRP^Trpv1^ mouse model [17,24]. Activation of TRPV1 with capsaicin permits transient and selective activation of AgRP neurons, leading to increased food intake, reduced energy expenditure [17,25], altered whole-body substrate utilization [26], enhanced ambulatory activity [26,27], and changes in foraging and repetitive behaviors [17]. This activation affects behavioral flexibility without impacting spatial learning [12], suggesting a specific role for AgRP neuron activation in modulating adaptive behaviors without affecting learning abilities. It is important to note that in this model, targeted channel expression in AgRP neurons is typically achieved by first performing a global knockout of the channel, followed by its selective reexpression in AgRP neurons. This method requires caution, as the initial global knockout may result in unintended phenotypes.

In addition to TRPV1, other chemogenetic tools include the inward rectifying potassium channel Kir2.1 and the bacterial sodium channel NaChBac (Figure 1). Kir2.1 can be exploited through its conditional expression in neurons, resulting in their chronic inhibition by hyperpolarizing the membrane potential. Conversely, NaChBac, when expressed in neurons, is employed to induce sustained excitation through depolarization. For example, chronic inhibition of arcuate GABAergic neurons using Kir2.1 has been shown to reduce aging-related weight gain [28]. NaChBac-mediated activation of POMC neurons did not reduce body weight, whereas Kir2.1-mediated chronic inhibition of POMC neurons led to obesity [29]. Moreover, NaChBac-mediated activation of arcuate leptin receptor (LepR) neurons, GABAergic neurons, or AgRP neurons has been shown to reverse leptin action on reducing type 1 diabetic hyperglycemia [30], while Kir2.1-mediated inhibition of arcuate GABA neurons produced leptin-mimicking rescuing effects [30]. 

These chemogenetic tools offer critical insights into neuronal regulation and behavior, complementing the applications of DREADDs. Nevertheless, expression of channels like Kir2.1 and NaChBac can lead to irreversible long-term cellular changes that may disrupt normal neuronal dynamics. Potential compensatory mechanisms in response to these alterations may further impact the accuracy and reliability of results. Additionally, the slower temporal resolution of these chemogenetic tools limits their effectiveness in capturing fast neural processes, making them less suitable for studies requiring precise temporal control.

### 2.3. Optogenetics

Optogenetics is a powerful technique for observing and controlling neuronal activity by using light to manipulate genetically-defined cells [31]. This technique involves genetically engineering targeted cells to express light-sensitive opsins, which can then be activated by light of a specific frequency to promote a conformational change in order to ultimately promote neural activation, such as with channelrhodopsin (Figure 1), or silencing, such as with halorhodopsin [31]. Through the use of transgenic animals, viral vectors, or a combination of the two, cell type-specific expression can be achieved [31]. For instance, injecting a Cre recombinase-dependent viral vector into the ARC of AgRP-Cre mice rendered AgRP neurons photo-excitable by expressing the light-activated cation channel channelrhodopsin-2 (ChR2) [8]. Optogenetic stimulation of AgRP neurons rapidly evoked voracious food consumption, with eating initiated immediately upon stimulus onset and terminated after its offset [8]. Additionally, optogenetic tools enable the specific manipulation of neural projections within targeted brain regions, allowing for precise control over neural circuits. For instance, optogenetic inhibition of the AgRP neuron projections in the paraventricular nucleus of the thalamus (PVT) impairs animals’ ability to associate spatial and contextual cues with food availability during food seeking [32]. Moreover, optogenetic manipulation of AgRP neuron projections in the dorsal lateral part of the dorsal raphe nucleus (dlDRN) regulated thermogenesis and energy expenditure without affecting appetite [33]. 

One of the key advantages of optogenetics is its spatial and temporal accuracy via targeted light application to specific brain regions [31]. This capability provides deep insights into how neural circuitry influences behavior. Optogenetic tools are particularly well suited for behavioral tests due to their ability to initiate targeted neuronal responses rapidly and precisely, offering a distinct advantage over chemogenetics. However, the surgical implantation of optical fibers presents a higher risk of complications, which can be challenging for inexperienced researchers. Additionally, light application can potentially induce tissue damage, so it is crucial to use appropriate intensity levels and include control groups in experiments. Moreover, the limited tissue penetration of light restricts optogenetic tools’ ability to modulate activity across large brain regions.

### 2.4. Targeted Cell Ablation

Targeted cell ablation is a powerful technique in feeding studies that allows researchers to selectively eliminate specific neuronal populations to investigate their role in regulating feeding behaviors. One potent cytotoxic agent used in targeted cell ablation is diphtheria toxin fragment A (DT-A), which enters the cell cytoplasm and inhibits protein synthesis, leading to cell death [34]. For example, to study the role of AgRP and POMC neurons in regulating energy homeostasis, researchers have expressed the diphtheria toxin receptor (DTR) (Figure 1) specifically in these neurons (AgRP^DTR^ and POMC^DTR^) [35]. Ablation of AgRP neurons in adult mice resulted in a rapid and significant decrease in body weight and a reduction in food intake while POMC neuron-ablated mice experienced a gradual increase in food intake and body weight [35]. Depletion of AgRP-expressing cells in adult mice also impaired food-seeking behavior [32] and decreased the fasting-induced elevation of circulating lysophosphatidic acid [36]. Interestingly, the neonatal ablation of AgRP neurons had minimal effects on feeding [37]. Additionally, the AgRP^DTR^ system allows for the selective and rapid ablation of AgRP neurons by injecting diphtheria toxin (DT) into brain regions that receive AgRP axon fibers. For instance, ablation of the PVT-projecting AgRP neurons impaired animals’ ability to associate spatial and contextual cues with food availability during food seeking [32], while ablation of the dlDTR projecting AgRP neurons resulted in a significant increase in thermogenesis [33]. Another powerful tool for targeted cell death is the use of caspase protease enzymes. Expression of caspase-3 effectively ablated AgRP neurons approximately 2 weeks after viral injection, resulting in blunted refeeding in fasted adult male mice [38]. 

The target cell ablation approach is valuable in feeding studies which allows for detailed exploration of how individual neurons or neuronal circuits contribute to various aspects of feeding and metabolism. However, there are notable limitations. The irreversible nature of cell ablation can complicate the interpretation of long-term outcomes as possible compensatory mechanisms might arise in response to the loss of specific neuronal populations. Additionally, the potential for off-target effects can introduce confounding variables. Finding the ideal dose of toxins for effective ablation can also be challenging, with incorrect dosing potentially leading to incomplete ablation or unintended damage to surrounding tissues.

### 2.5. Conclusions

In summary, the methodologies discussed for studying feeding behaviors by manipulating the neuronal activities each offer distinct advantages and limitations, reflecting their specialized applications in neuroscience research. Chemogenetic technologies, such as DREADDs, provide relatively feasible control over neuronal activation, allowing researchers to explore the effects on feeding behavior with the time scale from hours to days. However, challenges such as the spread of receptor expression, ligand-related delays, and potential receptor desensitization must be addressed. Optogenetics, with its relatively precise temporal and spatial resolution, excels in dissecting real-time neuronal dynamics and offers promising avenues for behavioral research, although its application is constrained by technical complexities and limitations in tissue penetration. Alternative methods like targeted cell ablation offer valuable insights, but the effect is not reversible in most cases and the potential off-target effects also require careful consideration. By integrating these methodologies and addressing their respective limitations, researchers can develop a more comprehensive understanding of how these techniques contribute to specific research goals, while effectively compensating for the temporal and spatial constraints inherent to each approach.

## 3. Visualizing of Neural Activities During Feeding Behaviors

### 3.1. Genetically Encoded Calcium Indicators

Rapid fluctuations in intracellular free calcium are indicative of neural activity, making calcium imaging a powerful tool for monitoring neuronal activity. Genetically encoded calcium indicators (GECIs) are extensively used to report intracellular calcium dynamics [39]. Among them, the green fluorescent protein (GFP)-based GCaMP is the most widely utilized, renowned for its high-affinity calcium binding and robust signal-to-noise ratio [39]. GCaMP sensors are engineered to detect calcium influx during neuronal activity through a calcium-binding domain that induces a conformational shift, leading to increased brightness of the GFP chromophore upon calcium binding [39,40]. GCaMP has undergone continuous optimization including the latest version, GCaMP8 [41], which offers enhanced sensitivity and faster kinetics, further refining the precision of calcium imaging experiments.

### 3.2. Fiber Photometry: In Vivo Population-Level Recording

**Figure 2:**
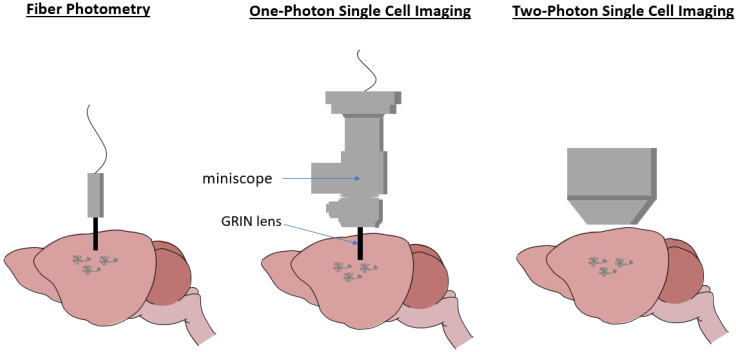
Methods used to visualize/track neural activity during feeding behavior.

Fiber photometry is an in vivo technique that monitors the population-level neural activity in free-moving or head-fixed animals. This method utilizes the selective expression of GECIs and flexible optical fibers (Figure 2), enabling the collection of a combined signal from a specific neuronal population while the animal is performing a behavioral task [42,43]. 

Researchers have employed fiber photometry to explore the role of AgRP neurons during feeding behaviors in free-moving mice. This technique reveals that food or sensory detection of food can rapidly reverse the activation of AgRP neurons induced by energy deficits, with a level of reversal correlated to food palatability and nutritional state [44]. Each bout of ingestion results in a transient dip in AgRP neuron activities [45]. Additionally, diet-induced obesity has been shown to reversibly attenuate AgRP neuron inhibition in response to food presentation [46,47]. Fiber photometry is also valuable for studying early postnatal development. By expressing jGCaMP7s in AgRP neurons of neonatal mice, researchers monitored calcium dynamics of AgRP neurons during the isolation and subsequent reunion of pups with their litter [48]. This approach revealed an increase in AgRP neuron activity throughout the isolation phase, while reunion with the litter caused a transient decrease in their activity, demonstrating a role of AgRP neurons in responding to disturbances in nest conditions [48]. 

Fiber photometry offers advantages such as being relatively low cost and easy to assemble [43]. It also enables simultaneous recording from multiple brain regions [49]. Nonetheless, it lacks cellular resolution, and distinguishing background autofluorescence from calcium signals can be challenging [42,50]. Additionally, the system requires mice to be tethered to the fibers during recordings, necessitating extra care and maintenance during long-term experiments [43].

### 3.3. One-Photon In Vivo Single-Cell Imaging

One-photon in vivo single-cell imaging is a powerful method for visualizing and recording the activity of individual neurons, typically performed using the miniaturized microscopes (miniscopes) equipped with a Gradient-Index (GRIN) lens (Figure 2). This method enables the simultaneous recording of hundreds of neurons over extended periods [50]. In particular, this approach has been used to track AgRP neuron activity in freely moving mice. In vivo calcium imaging revealed that 54 out of 61 AgRP neurons exhibited increased activity in food-restricted mice compared to ad libitum fed conditions [51]. The delivery of a food pellet to food-restricted mice resulted in reduced activity in 96% of AgRP neurons, with their activity gradually increasing but remaining slightly below the initial baseline after food removal [51].

Compared to electrophysiological single-unit recording and fiber photometry recording, one-photon in vivo calcium imaging offers the advantage of monitoring neural activity across large brain areas while preserving single-cell resolution in genetically defined neuronal populations [50]. This high resolution enables researchers to pinpoint the spatial distribution of neuron clusters [50,52]. Nevertheless, calcium imaging is constrained by the kinetics of calcium signaling, which limits temporal resolution relative to single-unit recordings [50]. Additionally, the surgical implantation of the GRIN lens can be challenging and may cause damage to nearby neural tissue [42].

### 3.4. Two-Photon In Vivo Single-Cell Imaging

Two-photon in vivo single-cell imaging is a highly advanced technique that allows researchers to visualize and record activity from individual neurons with exceptional resolution. In both head-fixed and free-moving animal models, two-photon microscopy offers the ability to capture detailed images of individual neurons by utilizing longer-wavelength, near-infrared light to excite fluorophores, making it ideal for studying neural dynamics in the deep brain. In head-fixed animals, this technique enables longitudinal tracking of neural activity during behavioral tasks through a cranial window [53] (Figure 2). For example, two-photon calcium imaging revealed that the responses of the glutamatergic neurons in the LH varied according to energy states in head-fixed mice when consuming randomly delivered sucrose rewards [54].

Meanwhile, after decades of effort, researchers have recently developed 2-photon microscopes and microendoscopes that can be carried on the head of freely moving animals [55,56]. These advancements are gradually making it possible to adapt two-photon imaging for free-moving animals, although this approach remains both challenging and in the early stages of development.

The longer wavelengths used in two-photon microscopy penetrates deeper into biological tissues and scatters less, which enhances the spatial resolution in the deep brain while minimizing photodamage and photobleaching. However, two-photon setups tend to be more expensive and technically demanding. Furthermore, two-photon imaging often has slower acquisition speeds compared to one-photon imaging, which can limit its ability to capture rapid dynamic processes. The large volumes of high-resolution data generated by two-photon imaging also present a challenge, as they are often complex and time-consuming to analyze.

### 3.5. Conclusions

In conclusion, regarding the techniques for visualizing and tracking neural activities during feeding behaviors, the in vivo cell imaging offers valuable insights with their own strengths and limitations. Fiber photometry is a relatively simple and less technically demanding technique, well suited for long-term recordings over extended periods without causing significant tissue damage or fluorophore bleaching. Yet, fiber photometry lacks cellular resolution and can be hindered by background autofluorescence. On the other hand, one-photon and two-photon single-cell imaging allows for the monitoring of neural activity over extended periods and across relatively large brain regions with single-cell resolution. However, these techniques are more expensive and technically challenging. Together, these methodologies represent powerful tools for visualizing the neural activities during feeding behaviors.

## 4. Analysis of Feeding-Associated Behaviors

### 4.1. Food Preference Test

**Figure 3:**
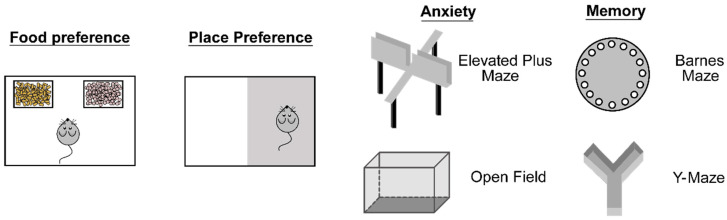
Methods used to analyze feeding-associated behaviors.

The food preference test is a behavioral assay used to assess an animal’s dietary choices when presented with two or more food options [57] (Figure 3). This test is widely used in feeding studies to explore how neural circuits and metabolic conditions, such as obesity or dietary restrictions, alter food choices. For example, researchers tested the preference for two differently flavored non-nutritive gels to investigate whether increased AgRP neuron activity generates a valence signal [51]. After the habituation of consuming both gels, ad libitum mice were conditioned to consume one flavor while undergoing optogenetic stimulation of AgRP neurons. Following this conditioning, AgRP^ChR2^ mice were presented with equal amounts of each gel to assess their food preference. Compared to the controls, AgRP^ChR2^ mice exhibited a reduced preference for the flavor previously paired with optogenetic stimulation, suggesting a role of AgRP neurons in eliciting a negative-valence signal [51].

Testing food preference is a simple yet powerful tool for uncovering fundamental feeding behaviors, making it accessible to researchers of any experience level. Of note, certain conditions must be met to ensure the validity of the experiment. First, it is essential to test consumers individually, as feeding biases can arise if subjects interact with each other during the trial [57]. Additionally, all food options should be presented in a consistent manner to each subject to ensure the accurate quantitative analysis of food consumption [57]. Finally, offering all food choices simultaneously, rather than sequentially, allows the subject to display a genuine preference, enhancing the reliability of the results [57].

### 4.2. Place Preference Test

Place preference tests are experimental paradigms used to assess an animal’s preference for specific environments or locations. In these tests, animals are placed in a two- or more-chambered arena where one or more areas are paired with certain stimuli (Figure 3). For instance, the light–dark box (LDB) test is a behavioral assay commonly adapted for place preference studies. This test consists of a box divided into two compartments: one brightly lit side and one dark side, which utilizes rodents’ tendency to avoid light to assess how anxiety levels affect animals’ choices [58]. Place preference tests are valuable in feeding studies as they help researchers understand the motivational aspects of feeding behavior, including how environmental cues influence feeding-related decisions.

Researchers have utilized the LDB test to investigate the role of subthalamic zona incerta (ZI) GABAergic neurons in inducing compulsive feeding [59]. In this study, photostimulation of ZI GABAergic neuron terminals in the PVT led to increased feeding on a high-fat diet even when the food was placed in the brightly lit chamber [59]. Additionally, given the association of binge eating with reward-system disorders [60,61], they demonstrated the motivational valence of this neural circuit by showing that optogenetic stimulation of ZI GABAergic neuron terminals in the PVT led to a prolonged time spent in the chamber associated with the stimulation, as measured by a two-chamber place preference test [59].

Analyzing behavior using place preference offers advantages such as a simple and quick setup, with subjects often requiring no prior training [58]. Importantly, the two or more arenas must be easily distinguishable to the subject [58]. Additionally, conducting a preliminary assessment of the subjects’ activity in the testing arenas is essential to appropriately pair each arena with specific stimuli. This preliminary step helps account for any inherent preferences or biases, ensuring a more accurate interpretation of the behavioral responses.

### 4.3. Anxiety Tests

When studying stress and anxiety-related behaviors, rodent-based behavioral assays are commonly used and can be conducted in various ways [62]. For instance, the elevated plus maze (EPM) offers subjects the choice of entering two opposing enclosed arms or two open, brightly-lit arms, with the entire maze elevated above the ground [62] (Figure 3). Shorter latencies to enter the open arms or longer time spent in these illuminated arms can be indicative of lower anxiety levels of the subjects [62]. Another useful assay for analyzing both anxiety and locomotor activity is the open field test (Figure 3). In this test, anxiety levels are assessed based on the latency to enter and the time spent in the exposed center of the field [62]. 

Researchers used the open field test with a novel object placed in the center to analyze novelty exploration and anxiety in subjects following the activation of AgRP neurons. Stimulation of AgRP neurons led to prolonged exploration of the novel object without affecting total activity, indicating a decrease in anxiety [17]. Additionally, the elevated plus maze was employed to investigate anxiety-related behaviors, where activation of AgRP neurons increased the time subjects spent in the open arms, further demonstrating that stimulation of AgRP neurons reduces anxiety [17]. 

While these behavioral tests are relatively straightforward to construct, replicating and interpreting the results can pose significant challenges. For one, the subjective nature of these studies can make replication difficult. Moreover, the “anxiety-like” behaviors observed in rodents may not fully capture the complexity of human anxiety [62]. Since these tests often rely on anthropomorphic projections of human traits onto animal behaviors, their relevance to human anxiety remains a point of debate, potentially limiting their applicability to human conditions.

### 4.4. Memory Tests

Spatial learning and memory are crucial for animal survival, particularly for navigating their environment during times of caloric need. Two commonly used behavioral paradigms for assessing spatial memory are the Barnes Maze (BM) and the Y-maze [63] (Figure 3). A typical BM consists of an aversely lit circular table with holes distributed uniformly along the perimeter, with one hole leading to a dark escape chamber in which the subject can escape the illuminated environment [64]. This setup allows the assessment of spatial learning and investigation of cognitive and memory deficits [64]. Similarly, the Y-maze tests spatial memory by allowing subjects to explore three arms of a maze, evaluating their ability to recall previously visited areas and their natural inclination to explore previously unexplored arms [65].

For instance, the BM was used to investigate the impact of chemogenetic activation of AgRP neurons on cognitive performance in the absence of food [66]. Researchers monitored the latency and distance traveled to reach the escape hole by AgRP neuron-activated mice and control mice during the learning phase. They observed no statistical differences between the groups, suggesting that increased activity of AgRP neurons during a spatial memory task does not interfere with memory retrieval [66]. In contrast, the Y-maze test revealed that chemogenetic activation of AgRP neurons led to a reduced proportion of correct spontaneous alternations towards random choices [66]. This result indicates that increased activity of AgRP neurons can impair performance in working memory tasks in mice [66].

The Barnes Maze (BM) is beneficial due to its minimally stressful nature and reliance on natural motivation [63], although the equidistant arrangement of the holes creates an artificial environment that may promote serial exploration [64]. On the other hand, the Y-maze is a simple behavioral assay but requires significant handling of animals, which can unintentionally induce stress [63]. Overall, spatial memory maze-based tests are cost-effective and provide rapid results, but they can be space-consuming and challenging to analyze, often requiring specialized tracking equipments [63].

### 4.5. Conclusions

In conclusion, the various experimental paradigms offer a comprehensive view of how neural mechanisms influence feeding-associated behaviors. Food preference experiments provide insights into how specific neuronal activities impact food choice, revealing critical aspects of feeding behavior. Place preference tests explore how environmental factors and neuronal manipulations contribute to the decision-making processes. Anxiety-related assays, such as the elevated plus maze and open field test, offer valuable perspectives on how feeding-related neural circuits influence emotional states. Finally, spatial memory tests, including the Barnes Maze and Y-maze, shed light on how neuronal activity influences spatial memory and cognitive performance. While these behavioral assays are effective in revealing different facets of feeding-related behaviors, they come with specific limitations, such as potential biases, the challenges in replicating results, and the constraints in translating these findings to human conditions. Nonetheless, each method contributes uniquely to our understanding of the feeding-associated behaviors. As methodologies continue to evolve, combining various behavioral analyses with manipulation of certain neurons and advanced neuroimaging techniques will be essential for uncovering the intricate dynamics of feeding behavior and its underlying mechanisms.

## 5. Identification of Neural Circuitry for Feeding Regulation

### 5.1. Mapping Neural Circuits

Neuronal tracers are used to map synaptic connections across brain regions. Retrograde tracing techniques, for instance, allow researchers to trace neural pathways from their synapses back to the cell bodies. This can be achieved through various methods, such as applying retrograde beads and retrograde virus to specific brain nuclei. The origin of the projections can then be visualized using fluorescent microscopy.

### 5.2. Retrobeads

**Figure 4:**
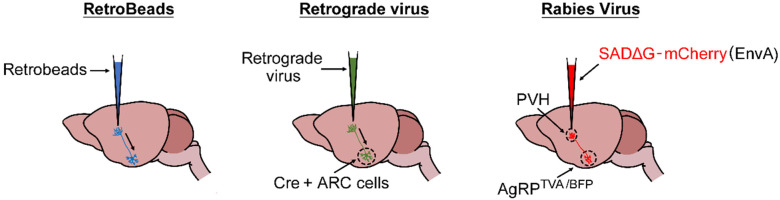
Techniques to investigate neural circuits important for feeding regulation.

Retrobeads are fluorescent latex microspheres, 30–90 nm in diameter, that offer long-term labeling due to their resistance to fading [67]. These non-toxic beads exhibit stable fluorescence with minimal diffusion from the injection site, making them suitable for long-term experiments [67]. However, retrobeads can be prone to clumping within the injection pipette [67], and are confined to labeling general populations of neurons rather than targeting specific neuronal groups within a region (Figure 4).

### 5.3. Retrograde Viruses

Retrograde viruses are valuable tools for genetically visualizing or manipulating neural projections. Naturally evolved viruses like the rabies virus, which spreads retrogradely, are neuro invasive but limited by their excessive virulence [68]. Alternatively, viruses such as pseudorabies (RPV) and adenoviruses can be delivered directly to the nervous system to infect neurons [68] (Figure 4). Additionally, recombinant adeno-associated viruses (rAAVs) can infect neuronal cell bodies at the exposure sites and be actively taken up by axons, facilitating retrograde access to projecting neurons [68] (Figure 4). When combined with a Cre recombinase system, rAAVs enable long-term transgene expression, which can be used to investigate the function of specific neural circuitry.

For example, researchers visualized AgRP neuronal organization and circuitry using a cell type-selective, axon-selective neuron transduction approach. Discrete axon projection fields, such as the PVH, were infected by an envelope protein A (EnvA) pseudotyped rabies virus vector, modified to express mCherry [SADΔG-mCherry(EnvA)], while AgRP neurons were engineered to coexpress the EnvA receptor, avian tumor virus receptor A (TVA), and blue fluorescent protein (BFP) [11]. Axonal transduction from the PVH resulted in high levels of mCherry and BFP colocalization in a subset of AgRP somas and axonal boutons within the PVH, while other AgRP neuron projection sites showed minimal mCherry expression [11]. This finding indicates prominent, one-to-one projections of AgRP neurons to the PVH.

Retrograde viruses offer the advantage of high levels of regional expression with relatively acceptable off-target effects [69]. When combined with specific genetic modifications, they can selectively target and label distinct neural populations, facilitating the detailed study of neural pathways. However, certain retrograde viruses, such as the rabies virus, are highly virulent and necessitate careful handling to mitigate risks to both researchers and animals [70]. Furthermore, factors such as virus titer, injection precision, and the health of the animal can influence the efficiency of viral infection and the accuracy of tracing, potentially impacting the reproducibility and reliability of results.

### 5.4. Validating Functional Connection

#### 5.4.1. In Vitro Optogenetic and Electrophysiology

Investigating functional neural connections in vitro using optogenetics and electrophysiology (ephys) assays involves optogenetic manipulation of upstream neurons or axon terminals in brain slices followed by recording the electrical responses in downstream neurons. For instance, researchers used optogenetic and electrophysiological methods to uncover the synaptic connection between AgRP neurons and the PVH [71]. Photostimulation of AgRP neuron axons in the PVH evoked inhibitory postsynaptic currents (IPSCs) in 29 of 61 PVH cells [71]. These responses were blocked by GABAA receptor antagonists, indicating that they were mediated by GABA [71]. With the same approach, an inhibitory projection was identified from the ARC tyrosine hydroxylase (TH) neurons to both POMC neurons and the PVH [72]. Photostimulation of ARC TH neurons evoked inhibitory postsynaptic currents (IPSCs) in 17 of 22 POMC neurons and 27 of 34 of PVH cells, which were largely attenuated by GABAA receptor antagonists [72].

#### 5.4.2. In Vitro Optogenetic and Behavioral Assays

Validating functional neural connections using in vivo optogenetics combined with behavioral assays allows researchers to precisely control and observe the impact of specific neural circuits on behavior. For example, researchers used projection-specific optogenetic stimulation followed by acute feeding assays to characterize the functional influence of different AGRP neuron projections in feeding regulation [11]. They identified multiple AgRP axonal projection fields, including the PVH and LH, that were sufficient to evoke feeding behavior comparable to AgRP neuron somatic activation, suggesting that AGRP neuron subpopulations form discrete, redundant, parallel circuits to control feeding behavior [11]. 

### 5.5. Conclusions

Overall, neuronal retrograde tracers, including retrobeads and retrograde viruses, are utilized to map synaptic connections. Retrobeads offer long-term labeling but face limitations such as clumping and non-specific targeting. Retrograde viruses, including the rabies virus and rAAVs, allow for the visualization of specific neuronal circuits, though they come with challenges such as pathogenicity and limited accuracy. Additionally, the in vitro optogenetic and electrophysiological methods, and the in vivo optogenetics paired with behavioral assays, offer a powerful approach to validate functional neural connections. Together, these methods provide crucial insights into the neural circuitry regulating feeding behaviors.

## 6. Discussion

In this section, we provide a critical evaluation of the methodologies used to study feeding behaviors in the hypothalamus (Table 1, Table 2 and Table 3). Each technique offers distinct advantages and limitations that impact their applicability and effectiveness across different research contexts. By assessing and highly condensing the strengths and weaknesses of these approaches, we aim to offer an informed perspective on how they can be optimally utilized in feeding research.

## 7. Conclusions and Future Directions

Traditional techniques including lesion studies and pharmacological studies have long been powerful tools in studying brain functions, including those involved in feeding behaviors. Lesion studies involve selectively damaging specific brain areas to observe changes in behavior or function, providing insights into the roles of particular regions in the tested processes, allowing foundational research to establish causal relationships between brain regions and behavioral outcomes. However, lesion studies often result in the destruction of a broad area of tissue, and this lack of specificity obscures the precise interpretation of the mechanism underlying the observed behavioral changes. Traditional pharmacological studies are widely used in feeding research to understand the effects of various substances on feeding behaviors and related physiological processes. These studies provide valuable insights by revealing how compounds including specific neurotransmitters, hormones, or receptors influence appetite, satiety, and metabolism. However, they are limited by non-specific effects that complicate result interpretations. Another traditional but still widely used method is electrophysiology. Electrophysiological methods, such as single-unit recordings or multi-electrode arrays, provide real-time measurements of electrical activity in single neurons or small groups of neurons, offering detailed insights into neural activity including patterns of synaptic transmission, firing rates, and network interactions with high temporal resolution. However, electrophysiological methods are technically complex, have limited spatial resolution, and are generally confined to short-term measurements.

In contrast, modern methodologies offer more refined tools for investigating neural circuits. The chemogenetic and optogenetic manipulation of neurons enables the precise control of neuronal activity with high temporal and spatial resolution. Advanced imaging techniques using fiber photometry, one-photon, and two-photon single-cell imaging allow for the real-time monitoring of neural activity in freely moving animals. These techniques can target specific cell types and pathways, providing insights into the functional roles of distinct neural populations and their interactions. Behavioral assays facilitate the link between neural activity and actual behaviors and provide a direct measure of functional outcomes. Validating functional connections in vitro or in vivo allows for a deeper understanding of how specific neural circuits contribute to physiological processes and behaviors.

In summary, each approach provides unique insights into the complex neural circuits and mechanisms underlying feeding regulation with specific advantages and limitations. Traditional methods continue to offer foundational knowledge and validation, while modern techniques provide the ability to dissect complex neural circuits with unprecedented specificity. Future research should aim to integrate these techniques to create a more comprehensive understanding of hypothalamic control over feeding. 

## Figures and Tables

**Table 1:** Comparison of the ligands and advantages and disadvantages of methods used to investigate the activity of feeding-associated neurons.

	Chemogenetics		Optogenetics	Targeted Cell Ablation
	DREADDs	Other Channels		
Ligand	CNO, Compound 21, perlapine, DCZ, etc.	Capsaicin for Trpv1; none for Kir2.1 and NachBac	Light of specific frequency	DT for DTR; none for caspases
Advantages	Rapid and reversible manipulation of neurons; elicits relatively long-term effects	Rapid and reversible or irreversible manipulation of neurons; elicits relatively long-term effects	Acute and reversible of neurons or axon terminals with relatively high spatial and temporal accuracy	Robust and permanent elimination of neurons
Disadvantages	Restricted control over valence and duration Potential off-target effects of ligands and receptors; risk of desensitization and downregulation of receptors	Restricted control over valence and duration Potential off-target effects of channels; potential compensatory mechanisms	Requires surgical implantation of optic fiber Potential tissue damage; restricted size of manipulation region	Potential compensatory mechanisms; challenges in dose selection

**Table 2:** Comparison of methods used to visualize/track neural activities during feeding behavior.

	Fiber Photometry	One-Photon In Vivo Single-Cell Imaging	Two-Photon In Vivo Single-Cell Imaging
Advantages	Relatively simple and low cost; enables simultaneous recording from multiple brain regions	Single-cell resolution; relatively compatible with free-moving animals compared to two-photon imaging	Single-cell resolution; enhanced spatial resolution in the deep brain with minimizied photodamage and photobleaching
Disadvantages	Lacks cellular resolution; challenges in distinguishing background autofluorescence	Technically complex; lower spatial resolution and increased photodamage compared to two-photon imaging	Technically complex; restricted compatibility with free-moving animals; slower acquisition speeds compared to one-photon imaging

**Table 3:** Comparison of methods used to identify neural circuitry involved in feeding regulation.

	Retrobeads	Retrograde Adeno-associated Virus (rAAV)	Rabies Virus
Advantages	Non-toxic; long-term labeling	High specificity; lower virulence; stable expression	High specificity; detailed circuit mapping
Disadvantages	Tendency to clump in injection pipette; lack of specificity	Potential off-target effects	High virulence; relatively technically complex
